# Textile-Based Potentiometric Electrochemical pH Sensor for Wearable Applications

**DOI:** 10.3390/bios9010014

**Published:** 2019-01-16

**Authors:** Libu Manjakkal, Wenting Dang, Nivasan Yogeswaran, Ravinder Dahiya

**Affiliations:** Bendable Electronics and Sensing Technology Group, School of Engineering, University of Glasgow, Glasgow G128QQ, UK; Libu.Manjakkal@glasgow.ac.uk (L.M.); w.dang.1@research.gla.ac.uk (W.D.); n.yogeswaran.1@research.gla.ac.uk (N.Y.)

**Keywords:** pH sensor, e-textile, potentiometric, graphite, wearable system

## Abstract

In this work, we present a potentiometric pH sensor on textile substrate for wearable applications. The sensitive (thick film graphite composite) and reference electrodes (Ag/AgCl) are printed on cellulose-polyester blend cloth. An excellent adhesion between printed electrodes allow the textile-based sensor to be washed with a reliable pH response. The developed textile-based pH sensor works on the basis of electrochemical reaction, as observed through the potentiometric, cyclic voltammetry (100 mV/s) and electrochemical impedance spectroscopic (10 mHz to 1 MHz) analysis. The electrochemical double layer formation and the ionic exchanges of the sensitive electrode-pH solution interaction are observed through the electrochemical impedance spectroscopic analysis. Potentiometric analysis reveals that the fabricated textile-based sensor exhibits a sensitivity (slope factor) of 4 mV/pH with a response time of 5 s in the pH range 6–9. The presented sensor shows stable response with a potential of 47 ± 2 mV for long time (2000 s) even after it was washed in tap water. These results indicate that the sensor can be used for wearable applications.

## 1. Introduction

Recently there have seen a surge in the development of wearable and disposable devices for health-monitoring applications. The disposable health-monitoring sensors such as temperature, pressure, strain and electrochemical biosensors are attractive for hygienic reasons. Among various reported sensors, the electrochemical biosensors are appealing for wearable healthcare application owing to their ease of operation, fast response and excellent sensitivity in body fluids [1,2,3,4,5,6,7,8,9,10]. The current technology for the development of such disposable sensors often relies on the use of non- degradable polymers, which could have negative environmental impact. Such issues could be alleviated by developing sensors on the eco-friendly substrate. In this regard, the textile-based devices have received significant attention for various applications [11,12,13,14], including sensing [12,15,16,17], energy storage [18,19], energy generators [20,21] and wireless communication [22,23]. The textile-based electrochemical biosensors are particularly attractive for non-invasive monitoring of chronic diseases as they nicely conform to the body surface without any side effects such as skin irritation and loss of vital health data. In particular, the textile-based pH sensors [11] are interesting as they allow monitoring of physiological response through sweat analysis and could also give information about the metabolic activity of the body or diseases such as diabetes [24,25,26,27,28,29]. As an example, the pH value of a healthy person is in the range of 4.5–6.5, but patients with cystic fibrosis have alkaline sweat (up to pH 9) due to the defect in bicarbonate-reabsorption (H^+^-secretion) [29,30,31]. In fact, the pH of other body fluids can also be used to determine distinct types of cancers, the healing process of wounds, cell proliferation, blood glucose levels, and metabolic activities of body etc. [1,2,3,4,32].

The tremendous promise of textile-based devices for various applications has led to the investigation of a wide range of materials and methods for the fabrication of the sensors. Some of these works are summarized in Table S1 in the supplementary section. Recently, carbon-based materials and polymers have found applications in chemical and biosensor-based textile fibers. For example, by using the template method, cotton fabric-based electrochemical sensors have been developed to monitor the lactate (sensitivity 0.3169 mA mM^−1^ and detection limit of 0.3 mM) in saliva [33]. The sensor comprised of Ag/AgCl as reference electrode and graphite paste modified with Prussian blue acting as a working and counter electrode [33]. In another work, a new textile weaving technique implemented for the development of glucose sensor (sensitivity 0.1098 µA dL mg^−1^) in which carbon ink mixed with potassium ferricyanide has been used as working electrode. Even though much work on textile-based sensors reports on body fluid analytes monitoring, the electrochemical pH sensors are limited [11]. The majority of textile-based pH sensors are based on optical or calorimetric methods [34,35,36]. As compared to the optical/calorimetric based sensors, wearable electrochemical sensors have many advantages for the online monitoring of health, including their miniaturization, low cost, simple hardware requirements and reusability. The sensors can be fabricated on the garments and integrated with miniaturized electronics for wireless applications. For example, a potentiometric pH sensor with electrodeposited IrO_2_ on conductive fabrics shows good performance with a sensitivity of 47.54 mV/pH [14]. However, future wearable systems will require fabrication methods that are compatible with textile manufacturing processes [37,38,39,40]. To this end, the printing technology offers a unique identity for both sensors’ fabrication and textile processing [15,39,41,42,43]. An appropriate choice of the material is another critical requirement for wearable applications and in printed electronics. The choice of the material is determined by various factors such as its adhesion between the textile substrate, low temperature processing, chemical stability (for biosensors applications), fast response, high sensitivity and biocompatibility.

For wearable applications of sensors, one of the key issues of electrode fabrication is the lack of flexibility of the sensitive materials. Recently graphite-polyurethane (G-PU) based electrodes have been shown to have excellent performance for biosensors such as dopamine and pH sensors [4,44,45] and energy storage devices such as supercapacitors [46,47]. A G-PU paste based electrode shows excellent flexibility and stretchability [4,48]. In this work, we are reporting a thick-film G-PU (as sensitive electrode (SE)) and Ag/AgCl (as reference electrode (RE))-based potentiometric pH sensor realized on a textile substrate for wearable application. Thick-film sensitive and reference electrodes are printed on cellulose-polyester blend cloth. For an SE, the binders play a crucial role in the mechanical and chemical stability of electrode. When a G-PU composite reacts with a pH solution, an electrical double layer (*edl*) is formed at the interface between electrode-pH solutions. The change in electrical properties such as impedance and capacitance of the *edl* depends on several parameters such as the electrode materials, interface between electrode-electrolyte, type of electrolyte, temperature, etc. [49,50]. The formation of the *edl* affects the electrochemical measurements and varies with the pH value of the solution, leading to changes in the surface potential of the electrode. Variations in the pH value of the test solution leads to changes in the electro-chemical properties of the SE. Unlike other binders in thick-film technology, PU is also involved in the electrochemical reaction. The PU used in this work provides both the mechanical and chemical stability to the SE. As compared to conventional binders, the PU offers excellent mechanical strength, flexibility and shape-memorable properties to the electrodes. In addition, PU offers excellent ionic conductivity for electrochemical reaction of the electrode [51]. The soft domains in PU such as polyethylene glycol and polytetramethylene ether glycol units allows flexibility and stretchability for the SE. The oxygen atoms present in the urethane groups and the soft domain units enhance the electroactive surface area of the electrode. The wettability of the electrodes increases due to the polyethylene glycol and polytetramethylene ether glycol units present in the film. The PU, interacting through hydrogen bonding between the end of the urethane groups and through polymerization, enhances the mechanical bond to the textile substrate [52,53]. The crystalline properties of SE were measured by using a X-ray diffractometer (XRD). The surface morphology and the cross-section of layers of the electrode on the cloth was observed through the scanning electron microscopic (SEM) image. The surface roughness of the sensitive material was investigated through 3D scanning of surface profilometer. The electrochemical performance of the sensor was analyzed through potentiometry, cyclic voltammetry (CV), and electrochemical impedance spectroscopic (EIS) methods. The fabricated sensor exhibits a sensitivity of 4 mV/pH in the range of pH 6–9, which is significant for the sweat-monitoring application.

## 2. Materials and Methods

**Figure 1:**
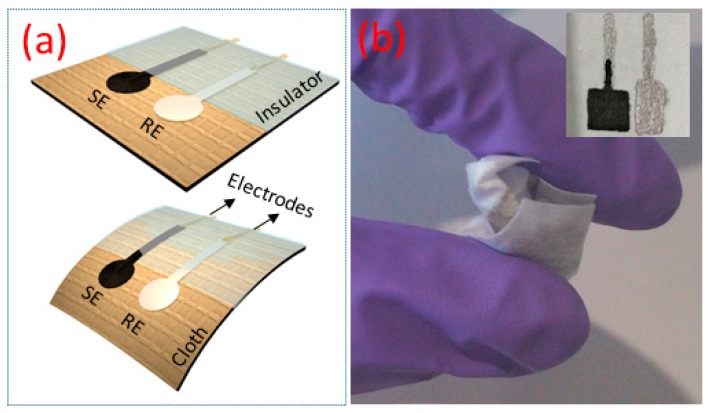
(**a**) Schematic representation of flexible potentiometric pH sensor (SE-sensitive electrode and RE-reference electrode) on cloth. (**b**) The image of flexible and crumpled pH sensor (inset shows the image of the electrodes).

The schematic representation of the potentiometric pH sensor developed on cellulose-polyester blended cloth is shown in Figure 1a. The cloth substrate used here consists of cellulose-polyester blend with 55% cellulose and 45% polyester and it has excellent wet and dry bidirectional strength due to the polyester content. The sensor comprises of Ag/AgCl reference electrode (RE) and G-PU SE deposited (area of 1 cm^2^) via screen printing. Initially, two strips (2 cm length and 2 mm width) of Ag conducting layers (RS components), one for SE and the other for RE, were printed on the top of the cloth and dried at 80 °C for 1 h. The SE was prepared by mixing 1 g of activated graphite powder (molecular weight of 12.01 g/m with particle size of <20 µm, Sigma Aldrich, St. Louis, MO, USA) in equal weight percentage of PU (1:1 wt %, PU resin from Blackfriar Paints) by using agate and mortar. The resulting composite was printed on substrate connected to Ag interconnects for an external circuit as shown in Figure 1a. Post-deposition the SE electrode was cured at 80 °C for 1 h in an oven. Ag/AgCl ink (Gwent Group, Wales, UK) was used as RE and is printed on top of the second Ag electrode and cured at a similar condition to SE. The image of electrodes for the potentiometric pH sensor on cloth is shown in the inset of Figure 1b. Finally, electrical connections between the sensors and measurement set up were made. After the external contact electrodes were soldered, a Polydimethylsiloxane (PDMS) ink was drop-casted as an insulative film to prevent the degradation of the electrical contact (Ag), when they are dipped in liquid solutions. The mechanical flexibility of the sensors is evident from Figure 1b.

The structural properties of the SE were investigated by using a Hitachi SU8240 field emission SEM. The surface morphology and roughness of the film were determined using a stylus profilometer (DektakXT™, Bruker, Billerica, MA, USA). The XRD, P’Analytical X‘Pert, with Cu Kα (λ = 1.514 Å) was used to investigate the crystal structure. The electrochemical sensing performance of the sensor was evaluated by using Autolab electrochemical workstation (PGSTAT302N, Metrohm, Runcorn, UK). We used two type of solutions for testing: (1) commercial pH buffer solution ranging from 5 to 9 (Sigma Aldrich), and (2) 0.1 M HCl and 0.1 M KOH solutions were prepared separately and were added dropwise into the distilled water for controlling the pH value of the solution. A commercial digital pH sensor (HI 98130 from Hanna^®^ Instruments, Leighton Buzzard, UK) was used to measure the pH value of this test solution. The sensing performance and the electrochemical characterization of the pH sensor were analyzed with potentiometric methods, CV and EIS in a two-electrode system. In the potentiometric method, an open circuit potential (OCP) is generated between the SE and RE and is used for measuring the Nernstian response. The analytical performance of the SE was investigated by CV analyses against a commercial glass-based Ag/AgCl RE (Sigma Aldrich) and the thick film RE in the potential range of −1 to 1 V at scan rate of 100 mV/s. The double layer formation and the electron/ion exchange at the SE with solution were studied by EIS analysis in a frequency range of 10 mHz to 1 MHz with an amplitude of 10 mV. Furthermore, as the sensor is proposed for a textile-based wearable health-monitoring application, the effect of washing is also studied. The cloth-based sensor was washed with tap water. After measuring the data with the sensor in solution with pH value 7, we dipped the sensor again in tap water and dried it at room temperature. The interference of glucose and urea were measured by mixing 2 mM concentrated glucose in phosphate-based buffer (pH 7.4 is PBS (phosphate buffered saline, PBS) is a balanced salt solution (1X) used for a variety of cell culture, ThermoFisher Scientific, Waltham, MA, USA) and similarly for urea 0.5 mM concentration of solution. Finally, the test solutions were prepared by mixing pH 7.4 with glucose and urea solution for testing the interference. The flexibility performance of the SE was investigated under cyclic bending condition at 11.40 mm bending radius up to 500 times.

## 3. Results and Discussion

### 3.1. Structural Properties

**Figure 2:**
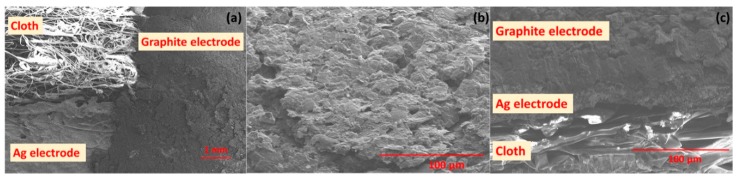
Scannng electron microscope (SEM) images: (**a**) top view of the sensitive electrode (SE) on cloth. (**b**) Surface morphology of the graphite sensitive electrode and (**c**) cross-sectional view of the layers of cloth, Ag electrode and graphite electrode.

The surface morphology of graphite and Ag conductive electrodes on the cloth substrate is illustrated in the SEM image of Figure 2a. The film shows a rough surface morphology with porous structure, which is very likely because the cloth is used here as the substrate. The surface of SE reveals that the film exhibits a layered like micro-flakes structure consisting of graphite, as shown in Figure 2b. The SEM images show a good interface between the graphite and PU which improves the mechanical strength of the film. The cross-sectional SEM image of the graphite and Ag thick film electrodes on a cloth substrate is shown in Figure 2c. The binder PU enables a perfect adhesion of film on the substrates and strong interaction between graphite flakes. The thickness of the Ag conductive electrodes is observed to be ~10 µm and for SE is ~100 µm from cross-section of SEM image. The surface morphology of the graphite electrode after cyclic bending (50 cycles and 100 cycles) were measured by using SEM and is shown in the supplementary information (Figure S1). The analysis shows that after cyclic bending, no significant change was observed in the structural morphology of the material.

**Figure 3:**
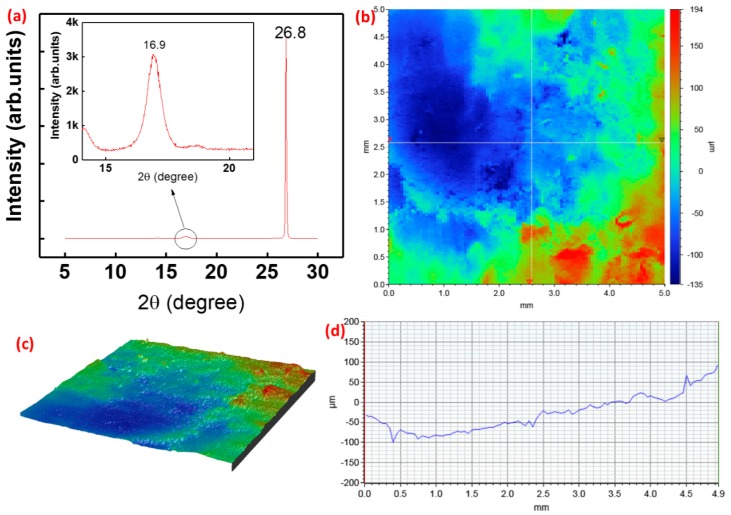
(**a**) X-ray diffraction (XRD) spectrum of the film (**b**) surface profile scanning of the graphite-polyurethane (G-PU) composite film over an area of 5 × 5 mm^2^ (**c**) 3D surface image of the sensitive electrode for roughness measurement. (**d**) Morphological scan on surface roughness.

The XRD pattern of the SE shows a strong (002) diffraction line of graphite at 2θ = 26.8° (in Figure 3a), which is in good agreement with those reported for graphite polyurethane composite [54]. The high intensity of the peak represent enriched amount graphite layers in the sensitive film. The prominent presence of the graphite leads to very good conductivity of the electrode. The conductivity of the electrode will enable the excellent formation of *edl* while reacting with pH solution, as discussed in the following section. In addition to this, the surface roughness has a strong influence on the electrochemical performance of the device due to varying surface area available for electrochemical reactions. The scanning, carried out in the area of 5 × 5 mm^2^ with a resolution of 50 µm/scan, is shown in Figure 3b. The 3D surface morphology of 5 × 5 mm^2^ of SE is shown in Figure 3c. The surface roughness of the film was measured by using a 3D profilometer. The average surface roughness was determined to be about 40 µm by using a profilometer, Figure 3d. To evaluate the variation of surface morphology due to the substrate used for printing of the electrodes, we also printed the electrode on the top of poly vinyl chloride (PVC) and measured the 3D profile, which is shown in Figure S2 in supplementary. As compared to the rough surface of cloth, the smooth surfaced PVC leads to reduced surface roughness of the material. The film shows a roughness of the 17 µm on the top of the PVC.

### 3.2. Electrochemical Properties and Sensing Performance

**Figure 4:**
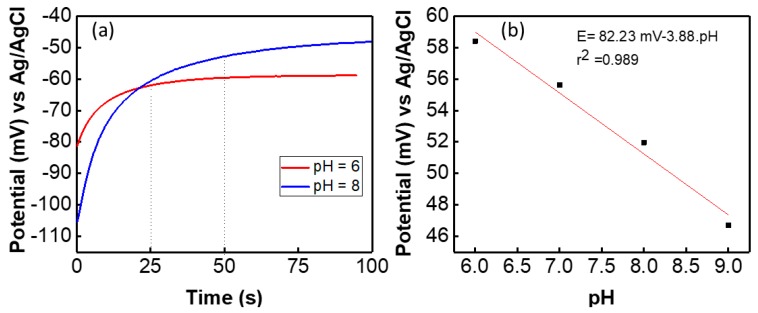
(**a**) Potential response of the sensor in pH buffer solution and (**b**) open circuit potential of the sensor with different pH value of solution.

Initially the potentiometric, CV and EIS measurements of the graphite SE versus glass RE were carried out by using two-electrode system. The results of the potential measurements of the graphite based pH sensors fabricated on cloth are shown in Figure 4. The stabilization time for a freshly prepared sensor is different for each pH solution. It was observed that when a solution contains more H^+^ ions the potential of the sensor stabilized faster than the solution that contains more OH^-^ ions. For example, in pH 6 the sensor stabilized in 25 s and for pH 8 the sensor reaches stabilization after 50 s, as shown in Figure 4a. The faster response for stabilization in pH 6 solution is because the H^+^ ions in the solution diffused faster than OH^−^ ions. The sensitivity of the presented cloth-based potentiometric pH sensor was measured at room temperature by dipping the sensor in buffer solution with different pH values. The OCP value of the sensor was measured and is shown in Figure 4b. As per Nernst equation, the potential difference between the sensitive and the reference electrodes is proportional to the solution pH [55]. The OCP between the graphite SE and glass-based Ag/AgCl RE was measured and found to be linear with ~4 mV/pH as the average slope (*n* = 4) of the OCP curve in the pH range of 5 to 9. The standard potential (E_0_) of the sensor is 82 mV with *R*^2^ = 0.989. Therefore, the slope factor (Figure 4b) called “sensitivity” is low ~4 mV/pH. As compared to pH sensitive metal oxides (for e.g., RuO_2_ and IrO_2_ [11]), the Nernstian slope of the graphite-based pH sensor is very low. For example, a RuO_2_-based sensor shows sensitivity of close to ideal Nernstian response 55 to 58 mV/pH [55,56,57,58]. The low sensitivity of this sensor could be explained by pH dependent surface charge of a conductive material [59,60]. As we mentioned in the introduction section, when the conductive graphite-PU is immersed into a pH solution an *edl* appears at the interface of the SE and the medium. The *edl* consists of diffuse layer and the Helmholtz layer. After *edl* formation, ion diffusion/specific adsorption and the charge transfer reaction takes place [59]. The ions from the pH solution approach the surface of the SE and then they are either attracted or repelled from the outer Helmholtz layer depending on the charges already present there. This interaction results in the formation of an electric bond and surface charge reactions. An ionic exchange process through diffusion takes place (as observed in the EIS analysis) on the surface of the electrode. The electrochemical properties of the *edl* formed on the surface of the graphite electrode vary with changes in the pH value of the solution. The concentration of buffer solution or the conductivity of the electrolyte can affect the *edl* properties and it may change the surface potential. For example, 0.1 M of pH and 1 M pH 7 may have different potential. Hence, the sensitivity of this conductive surface charge depends on parameters such as the electrode material/s, electrolytes, interface between SE-electrolyte, etc., [50]. An extensive study of the electrochemical properties of the graphite-PU SE has been carried out in the following section. Similar low sensitivity has been reported previously for pH-dependent surface charge of conductive electrodes in an electrolyte either due to protonation or deprotonation reactions or the specific adsorption of the dissociated water ions [59,60]. For example, the real sensitivity of the H-terminated diamond surface was found to be +5 mV/pH and the estimated sensitivity is 15 mV/pH due to preferential adsorption of water ions [60]. Similar low sensitivity was found for an organic semiconductor-based pH sensor [59].

**Figure 5:**
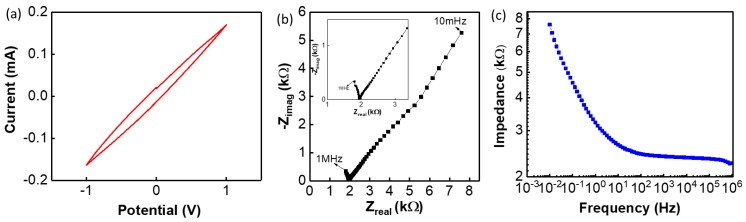
Electrochemical reaction of cloth and G-PU electrode: (**a**) cyclic voltammetry (CV) analysis for pH 7, (**b**) Nyquist plot (inset shows the high frequency), and (**c**) Bode impedance plot of the SE at pH 7 with a frequency range of 10 mHz to 1 MHz.

Further studies were carried out to investigate the sensing performance of SE. The CV analysis was carried out to analyse the electrochemical reaction of SE electrode and is shown in Figure 5a (pH = 5), at a scan rate of 100 mV/s against an Ag/AgCl glass RE. The CV analysis in Figure 5a is the characteristic curve of a carbon-based electrode. The electrochemical reactions of the SE with solution were analysed using EIS and the observed complex impedance is shown in Nyquist plot (Figure 5b and Figure S3 in supplementary). During the electrochemical reaction, the ionic diffusion occurs at the bulk of the electrode when the solution interacts with the surface of the SE. This ionic diffusion occurs mainly at the low-frequency range of the measurements, leading to the formation of double layer capacitance between the electrode and electrolyte as observed from the straight line in the low-frequency range. This is also confirmed in Bode impedance plot in the low frequency range, shown in Figure 5c. Subsequently, depending on the material property of the electrode, the generated charge moves from the electrode surface to the conductive layer. A charge transfer reaction is observed in the high-frequency range as shown in Figure 5b. In this range (>10 Hz) a reactance is observed on the electrode surface, as indicated by the straight line in the high-frequency range of the Bode impedance plot in Figure 5c. The incomplete semicircle is observed in the high-frequency range of the Nyquist plot (Figure 5b) is related to the charge transfer reactions on the surface of the electrode. From Figure 5b it was observed (in the high-frequency range, shown in inset of Figure 5b) that the electrode shows solution resistance of 1.8 kΩ. In addition to this, it has been proven that ion exchange processes took place in the SE surface, thus showing a capacitive reactance behaviour due to the separation and polarization of charges in the *edl.*

**Figure 6:**
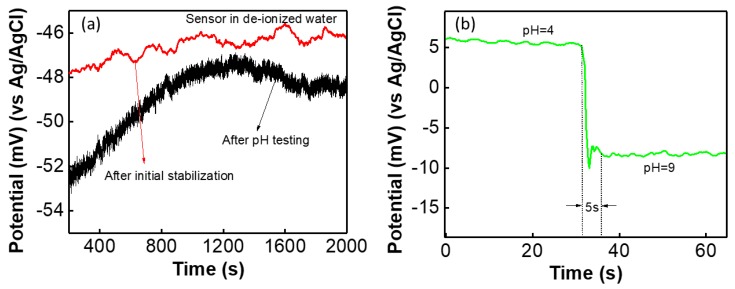
(**a**) Potentiometric cloth-based sensor performance in deionized water after initial stabilization and after measuring in pH solution. (**b**) Response time of the sensor in pH solutions.

For wearable potentiometric pH sensor application, both SE and RE were printed on the same cloth substrate. The sensitivity of the sensor is almost same for both thick film electrodes compared with the SE versus glass-based RE. After initial stabilization (48 h in distilled water) the potential difference between two electrodes, G-PU SE and thick film Ag/AgCl RE was measured for distilled water and is shown in Figure 6a. The sensor showed almost stable potential (~47 mV) for a long time with less than 1.5 mV variation in voltage. The sensor was washed in normal water and after washing the sensor showed a potential of 47 ± 2 mV for long time (for e.g., 2000 s) with only 1.5 mV deviation in potential. Furthermore, after repeated testing of the sensor in various pH solutions (range from 3–10), the sensor was washed in tap water and the OCP was measured in distilled water. The performance of the sensor is shown in Figure 6a. It was found that after initial reaction the sensor exhibited an almost stable potential. In both measurements, it was observed that the sensor shows small drift in potential in comparison with its performance in buffer solutions, as shown in Figure 5a. Furthermore, the sensor’s response time to change in the pH solution was determined by adding diluted HCl and KOH to the solution. The response time (t_90_) of the fabricated sensor is monitored by measuring its OCP reaching 90% of an equilibrium value after immersing it in solution. The sensor showed a fast response from acidic to basic solutions with a response time of 5 s, as shown in Figure 5b. This response time of the sensor is comparable with the metal oxide-based pH sensor [4,55]. We also observed that the sensor hysteresis (ΔV, change in voltage) is less than 0.5 mV and is negligible (as shown Figure S4 in the supplementary section).

**Figure 7:**
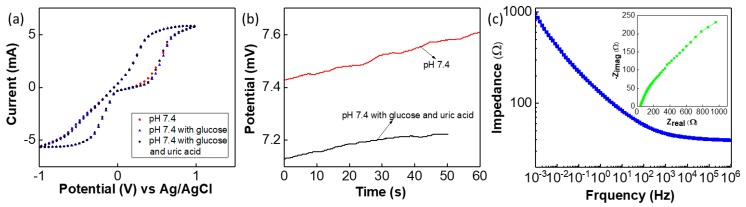
(**a**) CV analysis for pH 7.4, pH 7.4 with glucose and pH 7.4 with glucose and uric acid. (**b**) Open circuit potential (OCP) of the sensor in pH 7.4 and pH 7.4 with glucose and uric acid-based solution. (**c**) Impedance plot of the sensors (both SE and RE on cloth) at pH 7.4 based solution and inset shows the Nyquist plot.

As previously observed, the G-PU based sensor exhibits small interference with other ions (Na+ and K+ ions up to 100 mM concentration) and analytes including glucose [4]. In this work, the influence of other analytes such as glucose and urea often found in the body fluids on the performance of the sensor was evaluated via CV study. The CV response of the sensor to phosphate-based buffer solution (pH—7.4) is shown in Figure 7a. The influence of glucose on the sensor was evaluated by dissolving 2 mM of glucose in phosphate buffer solution, subsequently 0.5 mM of urea was added to the above solution to determine the influence of both glucose and urea on the sensor’s performance. The results of the CV study, shown in Figure 7a, indicate that these analytes have negligible influence on sensor’s performance. To further analyse this, an OCP value was measured for pH 7.4 and the solution with pH 7.4 alongwith glucose and urea (shown in Figure 7b). As compared to the CV curve on Figure 5a, the CV curve for the sensor (both RE and SE on cloth) in Figure 7a is different in shape. Similarly, the EIS plots shows that the impedance value of the sensor is lower as compared to the measurement for glass-based RE with SE as shown in Figure 7c even though the Nyquist plot have almost same shape (inset of Figure 7c). This may be due to the influence Ag/AgCl thick-film quasi RE. As compared to glass-based RE, thick-film RE exhibits lower impedance value and influences the sensor’s performance. The CV obtained under different scanning rate is shown in Figure S5 in the supplementary section. The influence of scan rate on CV response of the sensor in pH—7.4 buffer solution is shown in supplementary section. In a lower scan rate regime, we observed a significant redox peak. The dominant anodic and cathodic peak observed in CV may be due to the Ag/Ag+ redox reaction of the thick film quasi RE [39].

**Figure 8:**
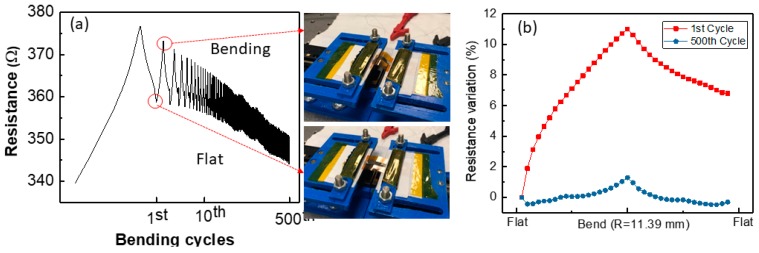
Cyclic bending of textile-based pH sensor (**a**) resistance monitoring of pH sensor until the sensor was bent up to 500 times, (**b**) comparison in pH sensor’s resistance variation between the first and 500th bending cycle when it was bent with a bending radius of 11.39 mm.

Finally, the electrical performance of the sensor under cyclic bending was investigated. The SE was bent up to the radius of 11.40 mm for 500 cycles and the electrical performance of the sensor in terms of its resistance was monitored, as shown in Figure 8a. As the number of cycles increases, the resistance drifts down. This may be due to the change in contact resistance between measurement wire and the SE. However, after 10 cycles of bending, the resistance tends to stabilize. Compared with the first and 500th bending cycle as shown in Figure 8b, the bending effect shows a 1% variation in resistance after 500 bending cycles but 11% at the first cycle.

## 4. Conclusions

The potentiometric thick-film pH sensor presented here is suitable for wearable applications, as evident from extensive analysis of the thick-film sensitive (graphite) and reference (Ag/AgCl) electrodes printed on cellulose-polyester blend cloth. Excellent adhesion and the surface morphology of the electrodes on the substrate are observed through the scanning electron microscopic image. 3D surface profilometry shows that the film has surface roughness of 40 µm and shows excellent crystalline property confirmed through XRD analysis. The fabricated sensor exhibits a sensitivity of 4 mV/pH in the range of pH 6–9. The response time of the sensor is 5 s and the results show negligible effect of interference and hysteresis. The sensor’s performance after washing in tap water is almost stable with a potential 47 ± 2 mV for a long time (2000 s). The cloth-based highly flexible pH sensor presented here has a fast response, but the sensitivity is low as compared to the ideal Nernstian response of other potentiometric pH sensors. Nonetheless, with the simple fabrication method presented here we are able to demonstrate that a pH sensor can be developed with methods that are compatible with textile processing. With further modification of the materials and integration method, we will improve the sensors’ performance in the future and also validate them for applications such as wearable health monitoring.

## Supplementary Materials

The following are available online at http://www.mdpi.com/2079-6374/9/1/14/s1, Table S1: Comparison of few textile based sensors performance. Figure S1: Surface morphology of the film (**a**) before bending (**b**) after 50 cycles of bending and (**c**) after 100 cycles of bending. Figure S2: (**a**) Surface profile scanning of the graphite-PU composite film over an area of 5 × 5 mm^2^ on the top of PVC substrate (**b**) 3D surface image of the sensitive electrode for roughness measurement. (**c**) Morphological scan on surface roughness. Figure S3: EIS analysis of sensor for different pH value of solution. Figure S4: Hysteresis of the potentiometric cloth based sensor for pH 9.8. Figure S5: Influence of scan rate on CV response of thick cloth-based sensor at pH- 7.4 buffer solution.
