# Supplementary material for: Textile-Based Potentiometric Electrochemical pH Sensor for Wearable Applications
Source: Biosensors (Basel). 2019 Jan 16;9(1):14. doi: 10.3390/bios9010014 (PMC6468877; doi:10.3390/bios9010014)
Supplement: Supplementary file 1 [file biosensors-09-00014-s001.pdf]

biosensors

MDPI

Supplementary Section

Table 1. Comparison of few textile based sensors performance.

Type of Sensors	Applications	Material	Sensitivity	Ref.
Chemical	DNT/TNT explosive detection	Carbon ink/GORE-TEX	DNT (0.38 µA mL µg^{-1}) and TNT (0.33 µA mL µg^{-1})	[1]
	Sweat sensor (sweat rate, pH, sodium sensor)	polyester/lycra; bromocresol purple (BCP); polypyrrole;	n/a	[2]
	lactate measurement in saliva	Carbon graphite paste modified with Prussian Blue (C-PB)/cotton fibre	0.3169 µA mM^{-1}	[3]
	Glucose measurement	Carbon ink/silk yarn	0.1098 µA dL mg^{-1}	[4]
	pH sensor	Conductive fabric/IrO_2	47.54 mV/pH	[5]
Physical	Motion detector	stainless steel/polyester fibre	n/a	[6]
	Strain sensor	Reduced graphene oxide/cotton fibre	bending radii (from 5 cm to 2.5 cm) increases from 16.7 kΩ to 167.9 kΩ	[7]
	Wearable keyboard	PEDOT:PSS/polyester fabric	n/a	[8]
	Temperature sensor	CNT-PMMA composite/yarn	0.75%/K	[9]

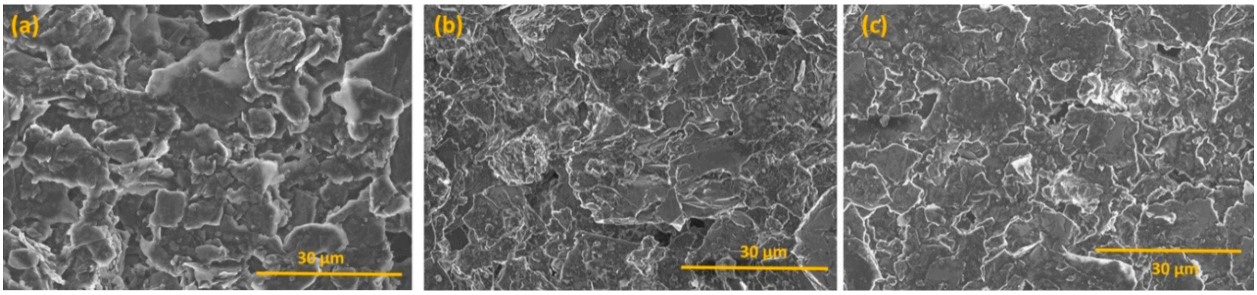

Figure S1. Surface morphology of the film (**a**) before bending (**b**) after 50 cycles of bending and (**c**) after 100 cycles of bending.

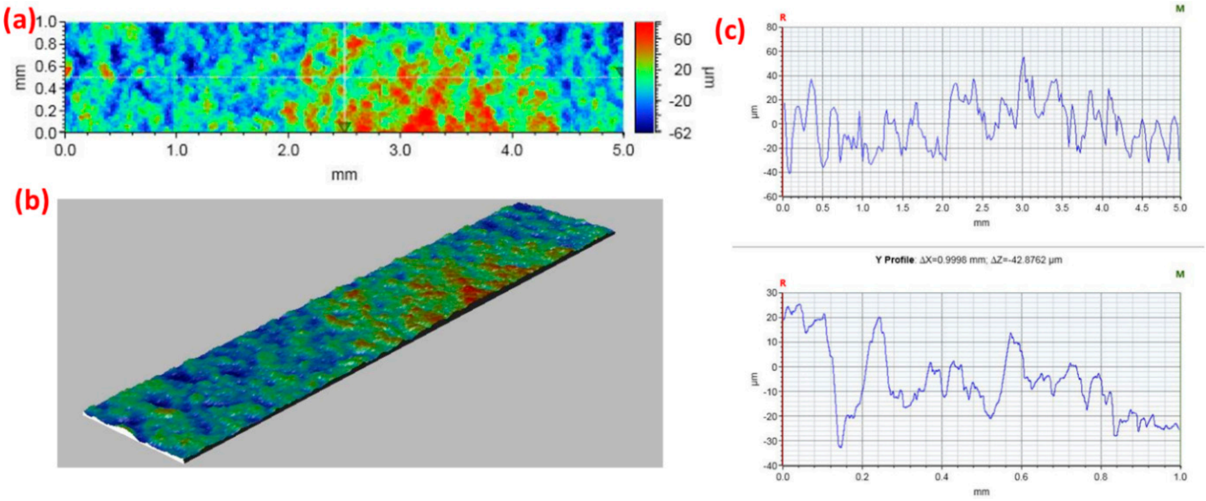

Figure S2. (**a**) Surface profile scanning of the graphite-PU composite film over an area of 5 × 5 mm^2 on the top of PVC substrate (**b**) 3D surface image of the sensitive electrode for roughness measurement. (**c**) Morphological scan on surface roughness.

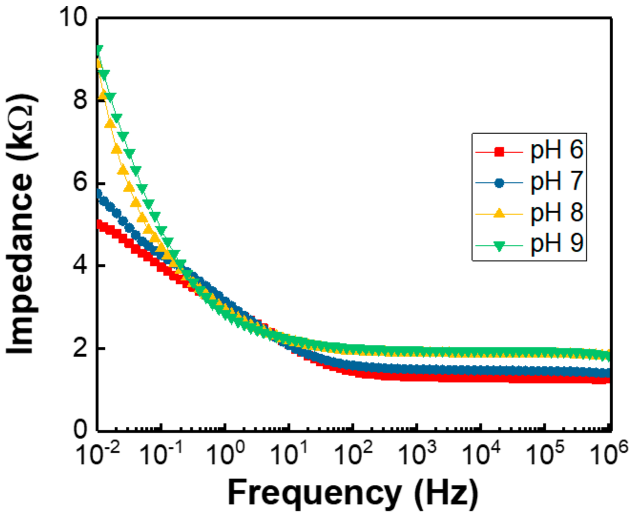

Figure S3. EIS analysis of sensor for different pH value of solution.

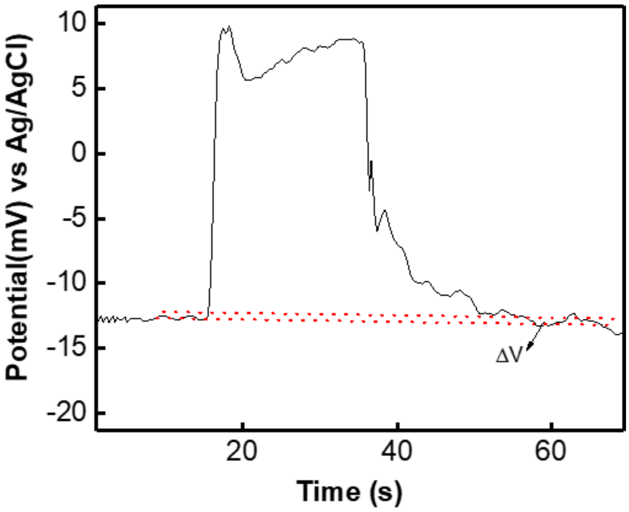

Figure S4. Hysteresis of the potentiometric cloth based sensor for pH 9.8.

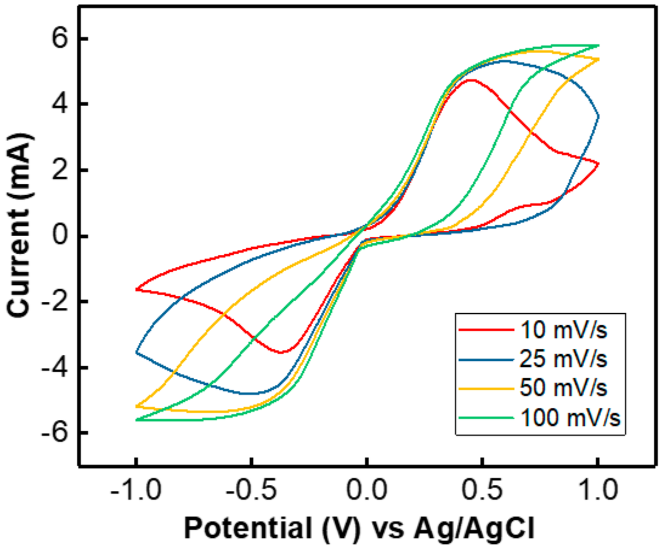

Figure S5. Influence of scan rate on CV response of thick cloth based sensor at pH- 7.4 buffer solution.

1. Min-Chieh, C.; Ray, W. J.; Padmanabhan, S.; Valdés, R. G.; Michal, G.; Tzu-Yang, C.; Joseph, W., Textile-based Electrochemical Sensing: Effect of Fabric Substrate and Detection of Nitroaromatic Explosives. *Electroanalysis* **2010,** *22* (21), 2511-2518.

2. Coyle, S.; Lau, K. T.; Moyna, N.; Gorman, D. O.; Diamond, D.; Francesco, F. D.; Costanzo, D.; Salvo, P.; Trivella, M. G.; Rossi, D. E. D.; Taccini, N.; Paradiso, R.; Porchet, J. A.; Ridolfi, A.; Luprano, J.; Chuzel, C.; Lanier, T.; Revol-Cavalier, F.; Schoumacker, S.; Mourier, V.; Chartier, I.; Convert, R.; De-Moncuit, H.; Bini, C., BIOTEX 2014;Biosensing Textiles for Personalised Healthcare Management. *IEEE Transactions on Information Technology in Biomedicine* **2010,** *14* (2), 364-370.
3. Malon, R. S. P.; Chua, K. Y.; Wicaksono, D. H. B.; Corcoles, E. P., Cotton fabric-based electrochemical device for lactate measurement in saliva. *Analyst* **2014,** *139* (12), 3009-3016.
4. Choudhary, T.; Rajamanickam, G. P.; Dendukuri, D., Woven electrochemical fabric-based test sensors (WEFTS): a new class of multiplexed electrochemical sensors. *Lab on a Chip* **2015,** *15* (9), 2064-2072.
5. Zamora, M. L.; Dominguez, J. M.; Trujillo, R. M.; Goy, C. B.; Sánchez, M. A.; Madrid, R. E., Potentiometric textile-based pH sensor. *Sensors and Actuators B: Chemical* **2018,** *260*, 601-608.
6. Kai, D.; Jianan, D.; Yunlong, Z.; Yi-Cheng, W.; Cheng, X.; Haiyang, Z.; Wenbo, D.; Yejing, D.; Bohong, G.; Baozhong, S.; Lin, W. Z., 3D Orthogonal Woven Triboelectric Nanogenerator for Effective Biomechanical Energy Harvesting and as Self-Powered Active Motion Sensors. *Advanced Materials* **2017,** *29* (38), 1702648.
7. Ren, J.; Wang, C.; Zhang, X.; Carey, T.; Chen, K.; Yin, Y.; Torrisi, F., Environmentally-friendly conductive cotton fabric as flexible strain sensor based on hot press reduced graphene oxide. *Carbon* **2017,** *111*, 622-630.
8. Seiichi, T.; Thomas, L.; Esma, I.; Atsuji, M.; Toshihiro, I.; G., M. G., Wearable Keyboard Using Conducting Polymer Electrodes on Textiles. *Advanced Materials* **2016,** *28* (22), 4485-4488.
9. Sibinski, M.; Jakubowska, M.; Sloma, M., Flexible Temperature Sensors on Fibers. *Sensors (Basel, Switzerland)* **2010,** *10* (9), 7934-7946.
